# Whole-genome single nucleotide variant phylogenetic analysis of *Mycobacterium tuberculosis* Lineage 1 in endemic regions of Asia and Africa

**DOI:** 10.1038/s41598-022-05524-0

**Published:** 2022-01-28

**Authors:** Thidarat Netikul, Yuttapong Thawornwattana, Surakameth Mahasirimongkol, Hideki Yanai, Htet Myat Win Maung, Virasakdi Chongsuvivatwong, Prasit Palittapongarnpim

**Affiliations:** 1 Faculty of Medicine, Siam University, Phet Kasem Road, Bangkok, Thailand; 2 Pornchai Matangkasombut Center for Microbial Genomics, Department of Microbiology, Faculty of Science, Mahidol University, Rama 6 Road, Bangkok, Thailand; 3 Department of Organismic and Evolutionary Biology, Harvard University, Cambridge, MA USA; 4 Department of Medical Sciences, Ministry of Public Health, Nonthaburi, 11000 Thailand; 5 Fukujuji Hospital and Research Institute of Tuberculosis, Japan Anti-Tuberculosis Association, Kiyose, 204-8533 Japan; 6 National TB Control Programme, Department of Public Health, Ministry of Health and Sports, Naypyitaw, 15011 Myanmar; 7 Epidemiology Unit, Faculty of Medicine, Prince of Songkla University, Had Yai, 90110 Thailand; 8 National Science and Technology Development Agency, Pathumthani, Thailand

**Keywords:** Tuberculosis, Bacterial genetics

## Abstract

*Mycobacterium tuberculosis (*Mtb*)* lineage 1 *(*L1*)* contributes considerably to the disease morbidity. While whole genome sequencing (WGS) is increasingly used for studying Mtb, our understanding of genetic diversity of L1 remains limited. Using phylogenetic analysis of WGS data from endemic range in Asia and Africa, we provide an improved genotyping scheme for L1. Mapping deletion patterns of the 68 direct variable repeats (DVRs) in the CRISPR region of the genome onto the phylogeny provided supporting evidence that the CRISPR region evolves primarily by deletion, and hinted at a possible Southeast Asian origin of L1. Both phylogeny and DVR patterns clarified some relationships between different spoligotypes, and highlighted the limited resolution of spoligotyping. We identified a diverse repertoire of drug resistance mutations. Altogether, this study demonstrates the usefulness of WGS data for understanding the genetic diversity of L1, with implications for public health surveillance and TB control. It also highlights the need for more WGS studies in high-burden but underexplored regions.

## Introduction

*Mycobacterium tuberculosis* (Mtb) is classified into 9 lineages^1–3^, with lineages 1–4 (L1–L4) being widely distributed around the world. Mtb L1, or the India-Oceanic lineage, is the only TbD1-positive Mtb lineage with high incidences in many high-burden countries around the Indian Ocean^4^. Although whole genome sequencing (WGS) is increasingly used for studying Mtb, our understanding of L1 genetic diversity and epidemiology in high-burden settings remains limited.

Mtb L1 was first classified into two main groups based on whole-genome SNPs, denoted L1.1 and L1.2, with five sublineages, denoted L1.1.1, L1.1.2, L1.1.3, L1.2.1 and L1.2.2^3^ and was subsequently expanded^5^. Recently, the scheme has been refined^6^ to consist of three main groups, L1.1-L1.3 and seven third-level sublineages, L1.1.1-L1.1.3 identical to the previous scheme, a novel L1.2.1, L1.2.2 equivalent to previous L1.2.1 and finally L1.3, equivalent to previous L1.2.2 and classified into L1.3.1 and L1.3.2.

Associations between each of these sublineages with geography have been studied. For example, L1.1.1.1 is predominant in Vietnam^7^, L1.1.3 is common in Myanmar^8^, Bangladesh^9^ and East Africa^10^ while L1.1.2.2 and L1.2.2 (previous L1.2.1) are associated with India and Southeast Asia, respectively^5^. Interestingly, around 80% of Mtb isolates in the Philippines, a high TB burden country, belonged to the last sublineage^11^. Thus, existing SNP-based classification schemes may not provide sufficient resolution for characterizing major sublineages in high burden countries such as the Philippines and India, each of which has several hundred thousand L1-infected patients each year^4^.

Spoligotyping is another widely used genotyping method, with data available from most countries. It evaluates the presence of 43 out of 68 genomic segments called direct variable repeats (DVRs) located in the CRISPR region, also called the Direct Repeat (DR) region^12^. This scheme classifies Mtb strains with deletions at DVR39–42, 44 and 48 as the East African Indian (EAI) genotype, which is equivalent to L1. Each spoligotype pattern is represented by a series of 0 s and 1 s indicating the absence or presence of each of the 43 DVRs, and is usually abbreviated into a 15-digit octal code with each number representing 3 DVRs sequentially for 14 digits with the last digit as either 0 or 1. Each spoligotype is given an identification number for the ease of use, called the spoligotype international type (SIT). For example, most of the L1.2.2 strains have SIT19 spoligotype or similar, collectively referred to as EAI2. Spoligotypes classify EAI2 into two groups, EAI2_MNL (Manila), associated with the Philippines, and EAI2_NTB (Nonthaburi), associated with Thailand.

Although spoligotyping is cheap and fast, its discriminating power is limited, and is confounded by the presence of common homoplastic spoligotypes such as SIT48 and SIT236, meaning that they could belong to one of several sublineages^5^. It was not known whether the same homoplastic spoligotype in different countries belonged to the same sublineage or not.

This study aims to refine the WGS SNP-based genotyping scheme of L1 to better understand its genetic diversity and population structure in high-burden regions. We performed phylogenetic analysis of 1,764 L1 samples, representing all major sublineages and endemic areas in Asia, Oceania and Africa. While this work was on-going, the revised nomenclature with three major sublineages had been proposed^6^. We adopt this three-group scheme here, but kept further nomenclature as backward compatible as possible. We compared the SNP phylogeny to the conventional spoligotype and the underappreciated 68-DVR patterns to gain deeper insights into the geographic distribution and dispersal history of L1 sublineages. We also examined genetic clusters and drug resistance mutations. The information is useful for evaluating TB burden and for understanding of the evolutionary history and pathogenesis, with implications for TB control.

## Results

### Phylogeny of L1 sublineages

WGS data of 1,764 M*. tuberculosis* (Mtb) L1 isolates from Asia, Oceania and Africa were obtained until November 2020. Most isolates were from Thailand (36%), Vietnam (26%), India (13%) and the Philippines (9%). The remaining were from other Asian countries (5%), Africa (n = 151; 9%) and Oceania (Australia and Hawaii; 2%) (Supplementary Table S1). Accession numbers of the raw paired-end reads of all Mtb isolates used in this study are provided in Supplementary Table S2.

**Figure 1:**
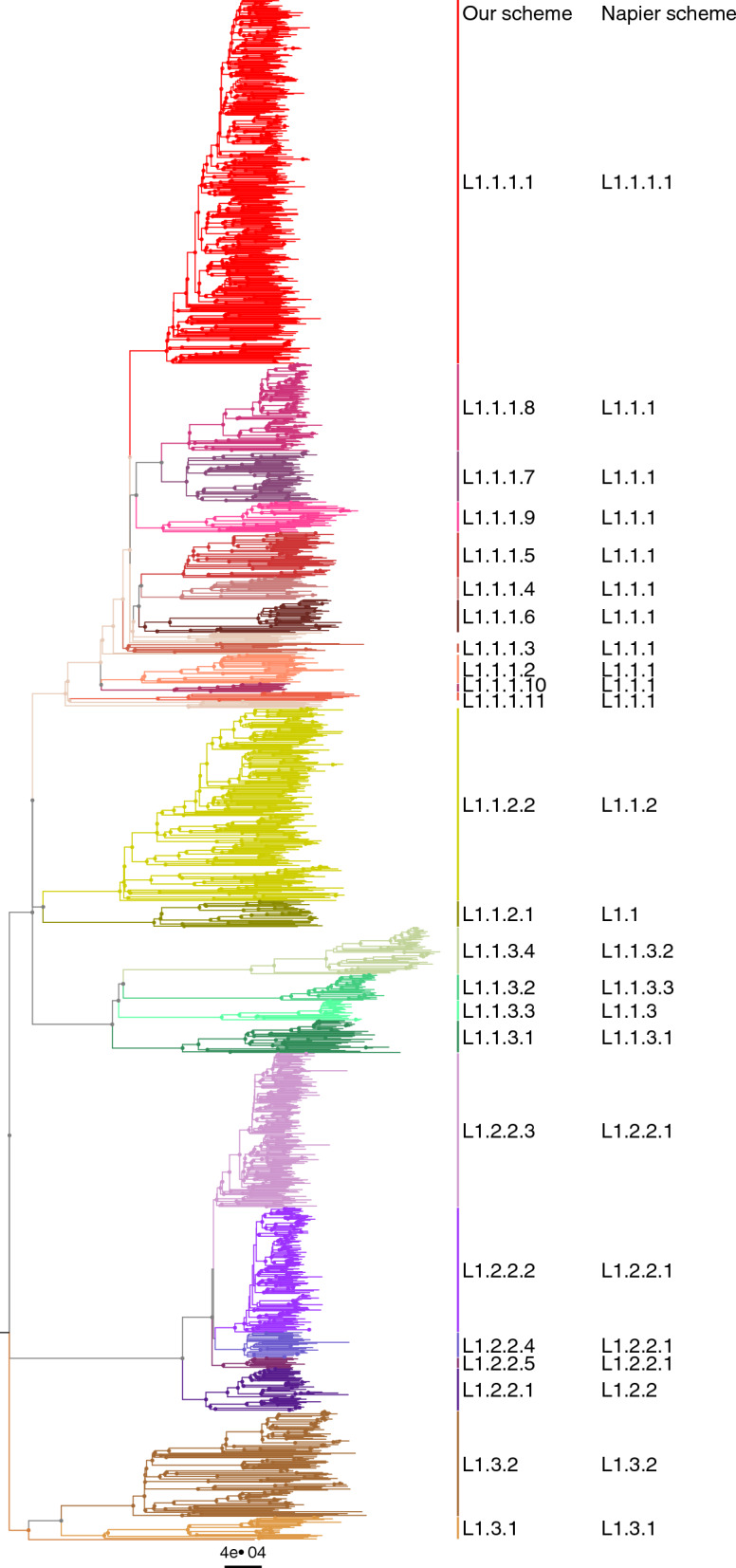
Maximum-likelihood phylogeny of 1,764 L1 isolates. Highlighting and left column indicate sublineages used in this study, right column indicates Napier's scheme^6^. An interactive online version is available in Microreact at https://microreact.org/project/ii9NYi9YWaWFjZkivV2JoC.

The phylogeny inferred from whole-genome SNPs supported the previously-described three major clades, L1.1-L1.3^6^, with subclades consistent with the existing schemes^5,6^ (Fig. 1).

L1.1 splits into three major sublineages, denoted L1.1.1–L1.1.3^3^. Most (97%) of L1.1.1 isolates were from Thailand or Vietnam. L1.1.1.1 was the most common sublineage of L1.1.1 (51%) and accounted for 85% of the samples from Vietnam but only 3% of Thai samples. L1.1.1.4 was also mainly found in Vietnam while most of the remaining sublineages were predominantly found in Thailand. We designated two new clades in L1.1.1: L1.1.1.10 from Vietnam and Thailand and L1.1.1.11 from West Africa^10^. About 3% of L1.1.1 isolates were left as unclassified (Fig. 1 and Supplementary Table S3).

L1.1.2 splits into two deep-branching clades^5^. L1.1.2.1 consists entirely of Thai samples while L1.1.2.2 is widespread across Africa, South Asia, mainland Southeast Asia (MSEA), and is most common in India (80%) (Supplementary Table S3). Notably, L1.1.2.2 was not identified in samples from East Asia or island Southeast Asia (ISEA).

L1.1.3 contains four distinct clades, designated as L1.1.3.1–L1.1.3.4 (Fig. 1). The first three have been reported in many Asian studies^5,8,13^. L1.1.3.4, was identified mostly among African samples (Supplementary Table S3), particularly in Malawi (69%). Here, the fourth numbering of L1.1.3 clades is consistent with Palittapongarnpim et al.^5^ but differs from the Napier’s scheme^6^, which does not recognize L1.1.3.3 (Fig. 1).

**Figure 2:**
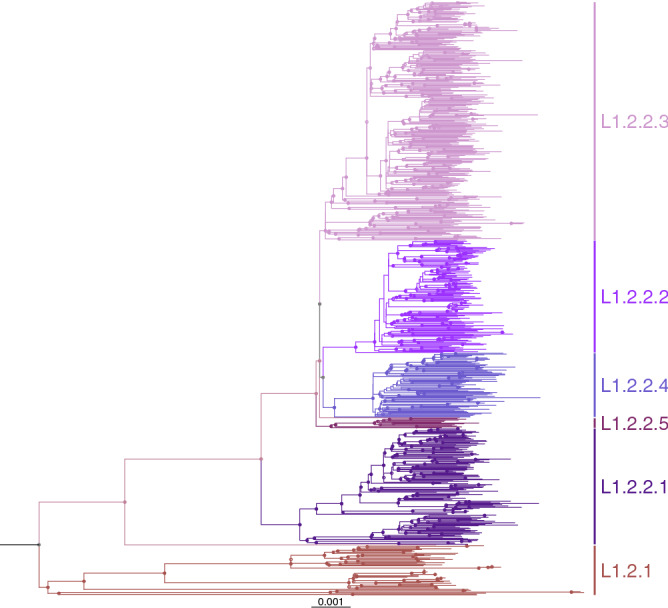
Maximum-likelihood phylogeny of 774 isolates of L1.2 obtained from a subset (*n* = 410) of the main dataset (1,764 isolates) and additional studies (*n* = 364) (Supplementary Table S2).

Interestingly, a sublineage in the Napier's scheme^6^ not present in our main dataset was L1.2.1, with most isolates being from patients in Europe. Therefore, we inferred a separate phylogeny of L1.2 isolates that included 410 isolates from our dataset and additional 364 isolates from Napier et al.^6^ and other recent studies^10,14^ (Fig. 2). L1.2 clearly splits into two major branches: L1.2.1 is a small clade (8.4% of L1.2) with mostly European samples while L1.2.2 is a large clade (91.6%) that is widespread across East Asia, MSEA and ISEA, consistent with Napier et al.^6^ (Table 2). Intriguingly, L1.2.1 contains a basal deep-branching clade of samples from East Timor and Papua New Guinea. L1.2.2 is classified into 5 sublineages, 1.2.2.1–1.2.2.5. L1.2.2.1–L1.2.2.3 were previously defined as L1.2.1.1–L1.2.1.3, respectively^5^. L1.2.2.1 was most common in Taiwan (31%), followed by the Philippines, Malaysia (Sabah) and Thailand (~ 10–15% each). L1.2.2.2 was mostly restricted to Thailand (89%). L1.2.2.3 was the largest sublineage (40% of L1.2). L1.2.2.4 was a small sister clade of L1.2.2.2. Both L1.2.2.3 and L1.2.2.4 were most common in the Philippines and nearby East Malaysia. L1.2.2.5 was the smallest sublineage, with most samples from Vietnam. The separation of L1.2.2 into five sublineages were supported by their comparable intragroup average pairwise SNP distances, which are less than the intergroup average pairwise SNP distances, and the fixation indices (Supplementary Fig. S2).

L1.3 (previously L1.2.2) was widespread in countries surrounding the Indian Ocean. It comprises L1.3.1 (18%) and L1.3.2 (82%) sublineages, with one basal isolate from Thailand. Almost all L1.3.1 isolates (88%) were from Eastern or Southern Africa, with a few basal isolates from India. In contrast, L1.3.2 was most common in India (36%), followed by Thailand (20%) (Supplementary Table S3).

### Analysis of 68 DVRs and spoligotypes

All L1 isolates had characteristic deletions of six DVRs, DVR39–42, 44 and 48, in the CRISPR region^5^. A small fraction (< 3%) of L1.1.1 isolates in many sublineages had only those six deletions (Supplementary Table S4). The corresponding 777777777413771 (SIT236) spoligotype was expected to resemble that of the most recent common ancestor of L1 (L1-MRCA). As the CRISPR region evolves primarily by deletion, sublineages with more deleted DVR blocks are expected to be more derived. We found that L1.1.1 had the lowest number of deleted DVR blocks, 4 on average, followed by L1.3.2 (4.5), L 1.1.2 (5), L1.1.3 (6), L1.2.2 (6) and L1.3.1 (7) (Supplementary Fig. S4).

Some sublineages correlated well to a unique set of DVR deletions that may be used as markers (Table 1). For example, L1.1.3 isolates share DVR33 and DVR56 deletions. L1.1.3.1 contained a clade with additional deletions of DVR6–7, DVR51 and DVR57, corresponding to spoligotype 777777757413371 (SIT292). L1.1.3.4 had additional deletions of DVR7–10, DVR12–19 and DVR34, corresponding to spoligotype 700777747413771 (SIT129). All L1.2 isolates shared DVR10 deletion. Most L1.2.1 had additional deletions of DVR11 and DVR33, except for a few basal isolates. Since DVR10–11 deletions were not used for spoligotyping, some L1.2.1 and L1.1.3 isolates do have the same spoligotype, 777777757413771 (SIT591), as a result of DVR33 deletion. Additional deletions of DVR4 and DVR30–31 were specific to L1.2.2 (typically 677777477413771, SIT19). L1.2.2.2 had an additional deletion of DVR17–35 (typically 674000003413771, SIT89).

**Table 1:**
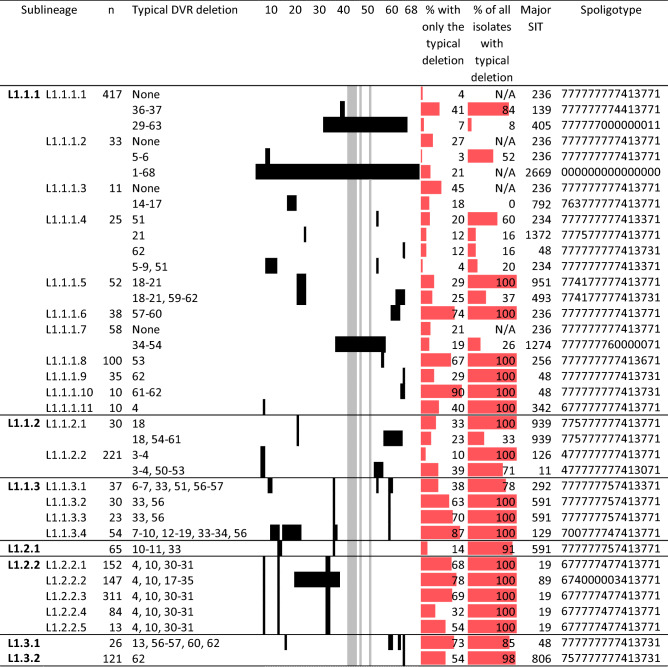
Typical DVR deletions of some sublineages. Deleted positions were mapped for all 68 DVRs, shown in the middle column. The signature deleted DVR of L1, DVR39-42, 44 and 48, is represented in grey; other deletions are in black. For each sublineage, the percentages of isolates having the typical DVR deletion exactly and of all isolates having the typical deletions with or without additional deletions are shown in red bars. The SITs and spoligotypes of isolates with only typical deletions are indicated in the last two columns.

Several sublineages shared a single DVR deletion block, e.g. L1.1.1.5 (DVR18–21), L1.1.1.6 (DVR57–60) and L1.1.1.8 (DVR53) (Table 1 and Supplementary Fig. S1). Nevertheless, those deletions could occur in other sublineages as well, limiting their potential use as reliable markers. Deletions of some DVRs were clearly homoplastic, occurring several times independently in the evolutionary history of L1. DVR62 is a characteristic of both L1.3 and L1.1.1.9, and is deleted together with DVR61 in L1.1.1.10. This deletion was also found in many other sublineages of L1.1.1. Thus, DVR62 deletion by itself is of limited genotypic value.

Several DVR deletions appeared to occur sequentially along the phylogeny. For example, most of L1.1.1.1 (*n* = 349, 84%) belonged to a clade with shared DVR36–37 deletions. The rest (16%) had intact DVR36-37 and were more basal to this clade, with 16 isolates having only six DVR deletions similar to L1-MRCA (Supplementary Fig. S1 and Supplementary Table S4). Within the DVR36–37 deletion clade, there were two subclades with additional deletions of DVR23 (*n* = 25) or DVR29–63 (*n* = 35).

Extreme deletions resulted in the complete absence of DVRs in 7 isolates from Thailand, all belonging to L1.1.1.2 (Table 1, Supplementary Fig. S1, and Supplementary Table S4). The WGS of the isolates had the read depth of 15–46 (median = 26) and the breadth of coverage at the sequencing depth of 20 between 23–96% (median = 74%). These isolates formed two separate clades, suggesting two independent events of complete deletion. The spoligotype-negative event was related to gene *cas1*, the deletion of which was associated with more vulnerability to DNA damage^15^.

### Geographic diversity of L1

**Figure 3:**
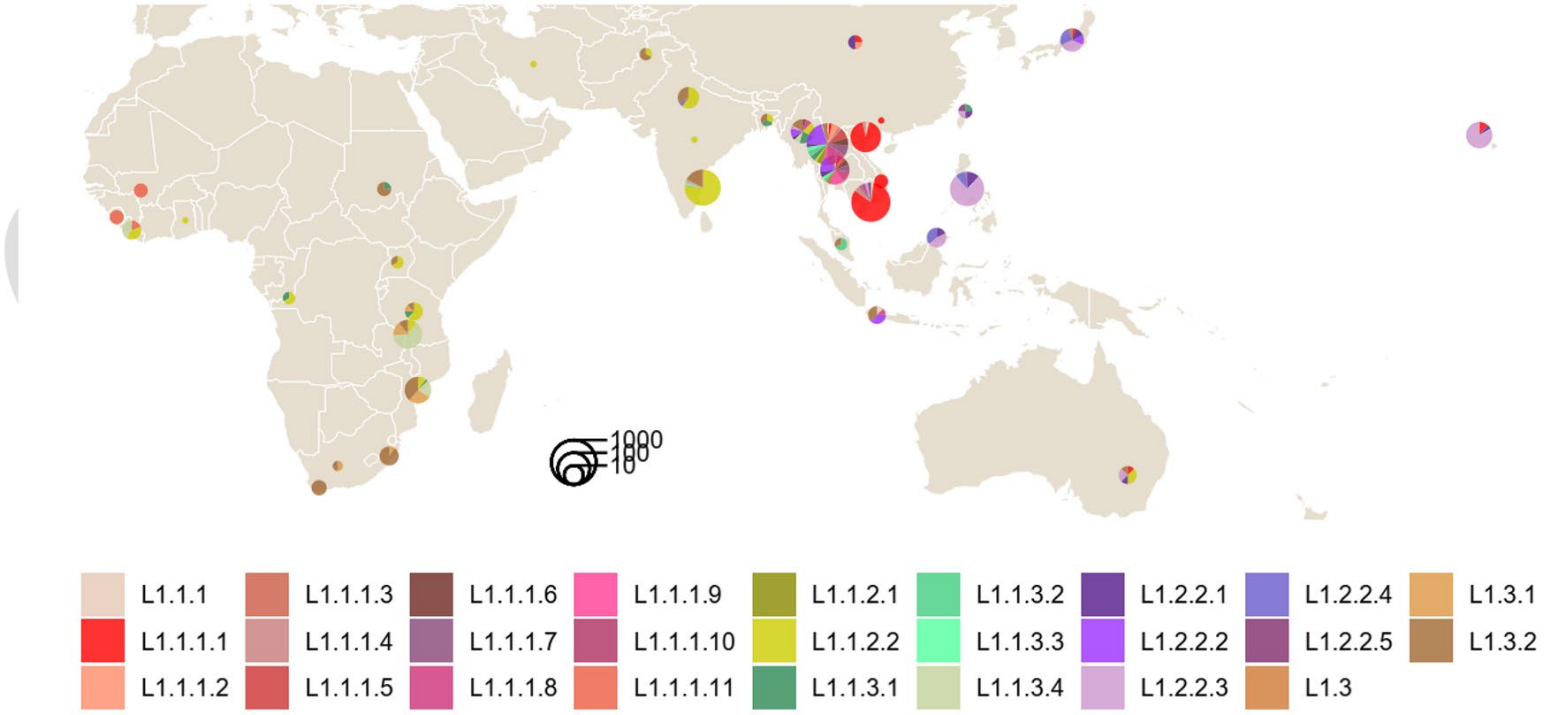
Geographic distribution of 1,764 L1 sublineages across Asia, Africa and Oceania. Pie sizes are proportional to the total number of isolates from each location. The map was created using the R package ggplot2 v3.3.5 (https://ggplot2.tidyverse.org).

Sublineages of L1 distributed preferentially around the Indian Ocean and the Western Pacific region (Fig. 3). L1.2 was predominant in ISEA and was rarely reported from Africa while L1.1.1.11 was restricted to West Africa^10^. Only L1.1.2.2 and L1.3 were distributed widely across Asia and Africa, particularly in South Asia.

At the country level, some countries, with > 50 samples, had a distinct predominant L1 sublineage: L1.1.1.1 for Vietnam, L1.1.2.2 for India, L1.1.3.4 for Malawi, L1.2.2.1 for Taiwan, L1.2.2.3 for the Philippines and East Malaysia, L1.2.2.2 and L1.1.1.8 for Thailand (Fig. 3, Table 2, and Supplementary Table S3). India, Thailand and Myanmar were the only countries in this dataset that harbored isolates belonging to all five common sublineages of L1, despite only 24 WGS samples from Myanmar.

**Table 2:** Distribution of 774 L1.2 isolates by country and sublineage. The 410 L1.2 isolates from the main dataset were supplemented with 364 isolates from additional sources; see Supplementary Table S2.

Country	L1.2							Total
	L1.2.1	L1.2.2						
		L1.2.2.1	L1.2.2.2	L1.2.2.3	L1.2.2.4	L1.2.2.5	Unclassified L1.2.2	
China		3						3
India			1	2	1			4
Nepal				1				1
Indonesia			4					4
Japan		3	3	8	6			20
Malaysia	1	17	1	127	54		1	201
Myanmar		1	3	1				5
Philippines		22	1	125	17	2		167
Taiwan		47		1		2		50
Thailand		19	131	3	2			155
Vietnam		1	3	8	1	9		22
East Timor	5			1				6
Australia		1		2				3
Papua New Guinea	2			1	3			6
Tanzania	1							1
Djibouti	1							1
Madagascar							1	1
Somalia	2							2
Italy		1						1
Netherlands	29	6						35
Sweden	8							8
United Kingdom	8	7						15
Switzerland				1				1
Canada	2	20						22
USA		2		30				32
Mexico		1						1
Peru	6							6
Ecuador		1						1
Total	65	152	147	311	84	13	2	774

### Sublineage barcoding SNPs

We identified SNPs uniquely shared by all isolates within each sublineage as markers for sublineage identification (Supplementary Tables S5 and S6). Among the 1,835 sublineage-specific SNPs, ~ 80% were in non-essential coding regions or in noncoding regions. We recommend using the full set of SNP markers when possible. We also provided a subset of 125 barcoding SNPs, prioritizing synonymous SNPs within essential genes (Supplementary Table S6).

### Drug resistance mutations

We used TBprofiler to predict drug resistance based on known genetic markers^16^ (Supplementary Fig. S5). The majority of isolates (*n* = 1,272, 72%) did not possess any resistance conferring mutations and, therefore, were presumably pan-sensitive. Isoniazid resistance was most common (*n* = 348, 19.7%), followed by ethionamide (*n* = 201, 11.4%), streptomycin (*n* = 188, 10.7%), rifampicin (*n* = 160, 9.1%), ethambutol (*n* = 99, 5.6%), pyrazinamide (*n* = 66, 3.7%) and fluoroquinolones (*n* = 28, 1.6%) (Supplementary Table S7). Mutations conferring resistance to other drugs were found in less than 1% of the isolates. By classifying drug resistance profile based on the latest WHO recommendations (see Methods), we found that 13% (*n* = 224) of the isolates were resistant to one first-line drug (mono-DR) while 7% (*n* = 123) were MDR. About 1% of the isolates were pre-XDR. Only one isolate was XDR. Most rifampicin-resistant isolates were also resistant to isoniazid (*n* = 142, 89%).

The prevalence of drug resistance varied across sublineages (Supplementary Figs. S5 and S6) which were correlated with the geography (Fig. 3 and Supplementary Table S3). MDR isolates were most prevalent among L1.2.2.1 (18%), L1.2.2.3 (13%) and L1.2.2.4 (33%), sublineages common in the Philippines and Malaysia (Table 2). This was consistent with a previous report of 19% MDR-TB in the Philippines^11^ although the MDR incidence among new TB cases there was only 2% in 2012^17^. We caution that the prevalence of drug resistance may not be representative due to different sampling designs used by source studies.

Mutations associated with drug resistance were diverse. All rifampicin-resistant isolates had variants in the *rpoB* gene, with 31 distinct alleles associated with changes at 13 amino acid residues. For isoniazid resistance, 99% of the isolates (*n* = 344) had mutations in *katG* or *fabG1/inhA* involving 36 alleles, with *katG* Ser315Thr being most common (52%), followed by the -15C > T mutation in the *fabG1* promoter (38%), with only 9 isolates having both variants (Supplementary Table S7). Among the other two first-line drugs, we identified 44 alleles of *pncA* among 63 isolates conferring pyrazinamide resistance and 30 alleles in *embA*, *embB* and *embR* conferring ethambutol resistance.

A small proportion of isolates (*n* = 28, 1.6%) possessed mutations in *gyrA* or *gyrB* conferring resistance to fluoroquinolones, with Ala90Val and Asp94(Gly/Ala/Asn) in *gyrA* being the most common (*n* = 26). Among those isolates, 18 were qualified as pre-XDR and mostly belonged to L1.1.1 or L1.3.2. The only isolate identified as XDR belonged to L1.1.1.9 from Thailand. In addition to pre-XDR mutations, it also had an insertion in *Rv0678* at position 192 that made it likely to resist bedaquiline^18^.

### Genetic clustering

We identified genetic clusters based on the number of SNP differences being within a pre-specified cut-point (Supplementary Fig. S7). Using three cut-points at 5, 12 and 20 SNPs, there were 20 (1.1%), 132 (7.6%) and 251 (14.4%) clustered isolates respectively, with an average cluster size of 2.0, 2.1 and 2.4 (Supplementary Table S8). The clusters were distributed across most sublineages, but were most common among L1.3 isolates (31%) and L1.1.3 (23%), and were rare in L1.1.1 (9.6%).

## Discussion

Here, we provide an updated classification scheme of Mtb L1 based on phylogenetic analysis of WGS data of samples from L1 endemic regions. The major differences between our nomenclature and the Napier's scheme^6^ are in L1.1.2, L1.1.3 and L1.2.2 sublineages (Fig. 1). Several sublineages associated with a specific country or region are recognized in our scheme but not in the Napier's scheme. For instance, L1.1.2.1, L1.1.3.3, L1.2.2.2 and several sublineages of L1.1.1 are mainly associated with Thailand. We identified a small deep-branching clade of L1.2.1 with samples from New Guinea and East Timor, suggesting an underexplored endemic area of L1.2.1 in ISEA. It is possible that a more complex population structure remains to be revealed as more WGS data become available.

**Figure 4:**
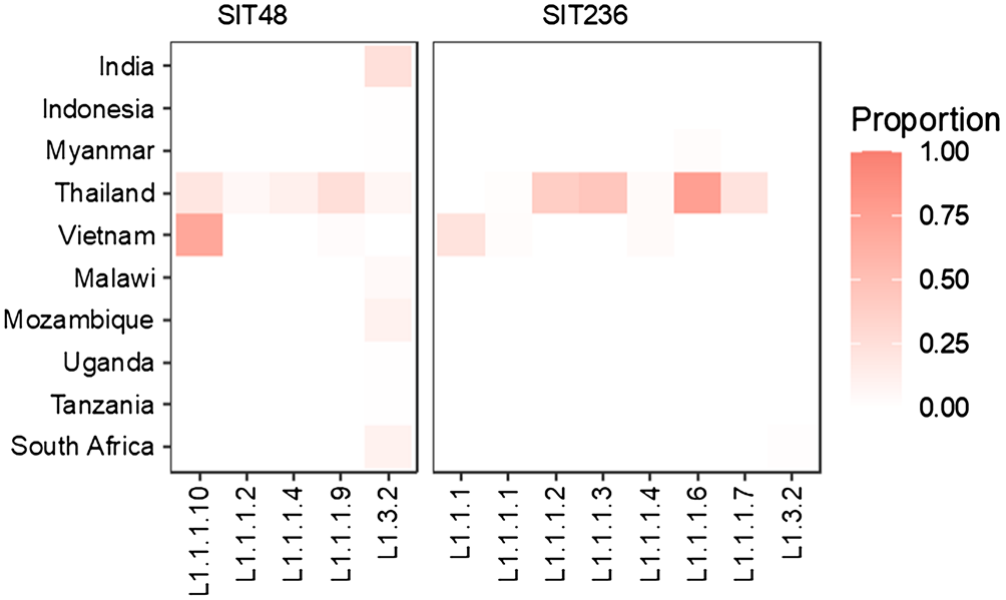
Proportions of isolates in each sublineage having SIT48 (777777777413731, EAI1) spoligotype (left) and SIT236 (777777777413771, EAI5) spoligotype (right) in various countries.

Comparisons between SNP sublineages and spoligotypes reveal some degree of congruence. There is greater congruence when the spoligotypes are defined by several deletion events, such as L1.1.3 and L1.2.2 (previously L1.2.1), which are equivalent to EAI6_BGD1 and EAI2 respectively. In contrast, spoligotypes defined by a single deletion event are more likely to be homoplastic. The most notable example is SIT48, typically defined by the deletion of DVR62, which occurred several times independently in the evolutionary history of L1. SIT48 can be a member of L1.1.1 or L1.3. Fortunately, since L1.1.1 is largely restricted to MSEA, SIT48 in other regions, such as India or Africa, most likely corresponds to L1.3 whereas SIT48 in MSEA may belong to either L1.1.1 or L1.3 as shown in Fig. 4.

We also clarify several genetic relationships between spoligotypes that could facilitate the development of better genetic groupings of spoligotypes. This is important as spoligotyping is more affordable in high-burden countries and provides data from much larger sample sizes with a higher geographic coverage than WGS.

The geography of isolates with most intact DVRs, similar to L1-MRCA, could be indicative of the region where L1 originated. Such isolates have the SIT236 spoligotype and are found exclusively in certain sublineages of L1.1.1, except for two L1.3.2 isolates (Fig. 4 and Supplementary Table S4). Since all sublineages of L1.1.1 except for L1.1.1.11 are mostly restricted to MSEA, where the diversity of L1 is also greatest (Fig. 3), it is possible that the L1-MRCA might have been present there. This is in contrast with recent genomic studies which predicted the origin of L1 in South Asia^10,19^, where none of the SIT236 isolates were present in our dataset. We note that their inferences were dominated by L1.1.2.2 and L1.3, and may suffer from small sample sizes (~ 300 or lower) and non-representative sampling.

One major limitation of this study is that it was based on WGS data collected for various purposes. Epidemiological findings such as the prevalence of drug resistance and genetic clusters were therefore only suggestive and require further investigation. A few source studies were intended for drug resistant bacteria which biased the estimates of drug resistance frequencies.

Our analysis revealed a vast diversity of resistance-conferring mutation combinations. The results confirmed the presence of the mutations in the rifampin-resistance determining regions (RRDR) in the vast majority (95%) of rifampin resistance isolates, which would allow the GeneXpert system to adequately detect rifampin resistance. Although available commercial line probe assays do not specifically identify mutations in about 37% of rifampicin-, 13% of isoniazid- and 55% of ethambutol-resistant isolates in this dataset, they should be able to diagnose the resistance by the absence of hybridization of wild-type probes. The line probe assay specifically detects most fluoroquinolone resistance mutations^20^.

## Conclusion

This study refines a genotyping scheme of L1, particularly of the L1.2 sublineages. Many basal isolates of L1.2 were recognized in ISEA samples, suggesting a possible study site to further elucidate the evolutionary history of the clade. Mapping spoligotypes onto the SNP phylogeny clarifies the genotypic identity of some homoplastic spoligotypes. Analysis of drug resistance mutations revealed a vast diversity of mutated alleles, particularly among the first-line drugs. This has implications for the utility of genetic tests for diagnosis of drug-resistant TB. Finally, genetic clusters in L1 are rare and small in size, suggesting a more limited transmission potential of L1, especially in comparison with L2^21^.

## Methods

### Dataset

We compiled a collection of whole-genome sequencing (WGS) data of 1,764 M*. tuberculosis* L1 isolates from countries in Asia, Oceania and Africa. This is the main dataset. Most of the data were publicly available while a small proportion was from unpublished studies. Supplementary Table S1 provides a list of data sources and references for the WGS data used in this study. A full list of accession numbers for all 1,764 isolates is in Supplementary Table S2. Supplementary Table S3 shows the distribution of samples across countries and sublineages. We also obtained additional 364 L1.2 isolates from three recent studies^6,10,14^. This set was analyzed together with the 410 L1.2 isolates from the main dataset, referred to as the L1.2 dataset (*n* = 364 + 410 = 774) (Supplementary Table S2).

### SNP calling

Short reads data were quality-trimmed using trimmomatic v0.39 (sliding-window trimming with window size of 4 and read quality threshold of 30)^22^. The processed short reads were mapped to the *M. tuberculosis* H37Rv reference genome (NC_000962.3) using bwa mem^23^. Duplicate reads were masked using Picard’s MarkDuplicates, and per-sample SNPs were called using GATK HaplotypeCaller v4.1.6.0 (base quality score ≥ 20, haploid model)^24^.

As additional sample quality control, we excluded samples if (i) the median read depth below 10 or the median breadth of coverage (depth at least 10) below 10%, or (ii) it was redundant as determined by the BioSample accession number and sample metadata if provided by the original study, or (iii) the processed reads had unusual %GC content, or (iv) SNPs specific to different sublineages were present in substantial proportions, indicative of mixed strains. The total of 1,764 isolates passed this sample quality control.

Joint SNP calling of all 1,764 isolates was performed using GATK GenotypeGVCFs v4.1.6.0^24^. For variant quality control, we dropped SNPs within a region annotated as repeat region, IS6110, PE, PPE, mobile element, phage or 13E12 repeat family. SNPs within known drug-resistant genes were excluded as in Ajawatanawong et al^25^. Finally, SNPs with quality by depth (QD) < 2 or root mean square of mapping quality (MQ) < 40 were also excluded. This resulted in 153,244 SNPs across 1,764 genomes. The additional L1.2 isolates were processed similarly.

### Phylogenetic analysis

SNP calls passing the quality filters were converted into a multiple sequence alignment using a custom script based on GATK VariantsToTable (available at https://github.com/CENMIG/snpplet). Point deletions and missing calls were converted into gaps in the alignment. The H37Rv strain (L4) was used as an outgroup. A maximum likelihood (ML) phylogeny was inferred using IQ-TREE v2.1.1^26^. The best-fit substitution model was determined to be GTR + G4 by ModelFinder^27^. The bootstrap branch supports were based on 1,000 replicates. We also inferred an ML phylogeny correcting for ascertainment bias as shown in Supplementary Fig. S8 and obtained consistent bootstrap support for all sublineages^28^.

### Population structure

Summary statistics of genetic diversity of each sublineage and genetic divergence between sublineages were calculated from the filtered SNPs using scikit-allele v1.3.1^29^. These included genome-wide averaged nucleotide diversity (π) for each sublineage, pairwise SNP difference and pairwise *F*_ST_ (using Hudson estimator^30^) for within sublineage and between sublineages, and principal component analysis (PCA) of genome-wide SNPs.

### Detecting direct variable repeat (DVR) deletions

To determine deletion pattern of the 68 known DVRs, we mapped the short-read data to a custom-made reference sequence that contained all the DVRs. This reference was constructed based on an L1 isolate (accession: ERR752247) with 62 DVRs, augmented with DVRs 39–42 from the H37Rv strain (accession: NC_000962.3) and DVRs 44 and 48 from an L2.1 isolate (accession: ERR718276).

Each DVR segment was identified as a deletion if one of the following criteria was satisfied: (i) the median read depth was below a cutoff and the proportion of sites with at least one read mapped to was < 50%; (ii) the 25% quantile of read depths was < 3; (iii) the 5% quantile of read depths was 0; (iv) if the overall median depth (across all DVRs) was > 50, the median read depth was smaller than a half of the median read depth. For (i), we used the cutoff value of 5, or half of the baseline median read depth calculated from all DVR regions, whichever was lower. This choice as well as the criteria (ii) and (iii) accounted for low-depth samples. The criterion (iv) accounted for samples with high sequencing depths by adjusting for overall read depth across all DVR regions. Given a DVR pattern, we extracted a relevant set of 43 DVRs to determine the spoligotype. SIT and spoligotype group for each spoligotype was obtained from the SITVIT2 database^31^. The code for DVR calling is available in GitHub at https://github.com/ythaworn/dvrcaller.

We tested our algorithm using a 480-isolate subset where spoligotypes had been determined experimentally and DVR patterns had been independently identified using a de novo assembly-based approach^5^. The above criteria yielded ~ 6% difference for the spoligotype and 16% difference for the DVR pattern. We also compared our predicted spoligotypes with two other methods, SpoTyping^32^ and Galru^33^ (Supplementary Table S2). The number of mismatches between each pair of methods ranged between 14%–17%. The difference in predicted spoligotypes was usually only about 1–2 DVR segments. Manual inspection of mapped read depths indicated that our method appeared to be more reliable.

### Revising genotyping scheme and identifying lineage-specific SNPs

We used the following criteria to delineate genotypes based on the inferred phylogeny (Fig. 1). First, we preserved the genotypes that have previously been defined. Sublineage of each isolate was identified using two existing SNP-based genotyping schemes for *M. tuberculosis* L1^3,5^. Second, we assigned a new genotype to a monophyletic clade of previously unclassified strains with at least 10 isolates, bootstrap support ≥ 90%. We also required that all isolates shared at least one SNP specific to the group that was absent outside the group. Third, we expanded the definition of existing sublineages to include more of previously unclassified strains if the final clade also had consistent genomic and epidemiological features such as the DVR pattern and geography, and satisfied the previous conditions.

Once genotypes have been defined, we identified a list of SNPs specific to each genotype using a custom script (available in GitHub at https://github.com/ythaworn/group-specific-variants). These identified sublineage-specific SNPs serve as markers for genotyping those sublineages. We annotated variants within an ORF using four states of essentiality: essential (ES), growth defect (GD), nonessential (NE) and growth advantage (GA)^34^. Supplementary Table S5 shows that number of sublineage-SNPs identified. The full list of annotated sublineage-SNPs is in Supplementary Table S6.

### Drug resistance mutations

We used TBProfiler^16^ to predict resistance of each isolate to the following 19 anti-TB drugs: rifampicin, isoniazid, pyrazinamide, ethambutol, streptomycin, moxifloxacin, ofloxacin, levofloxacin, ciprofloxacin, amikacin, kanamycin, capreomycin, ethionamide, para-aminosalicylic acid, cycloserine, linezolid, bedaquiline, clofazimine and delamanid. Classification of drug resistance type was according to the latest World Health Organization (WHO) recommendations as follows. mono-resistance (mono-DR): one first-line drug (isoniazid, rifampicin, ethambutol, pyrazinamide); polydrug resistance (poly-DR): more than one first-line drug other than isoniazid and rifampicin together; multidrug resistance (MDR): both isoniazid and rifampicin together; pre-XDR: MDR and any fluoroquinolone; extensive drug resistance (XDR): pre-XDR and at least one of bedaquiline or linezolid^35,36^.

### Genetic clustering

We identified genetic clusters as clades in the phylogeny containing isolates that can be linked via pairwise SNP distances of at most 5, 12 or 20 SNPs^37,38^.

## Supplementary Information

Supplementary Information 1.

Supplementary Figure 1.

Supplementary Figure 3.

Supplementary Figure 8.

Supplementary Tables.

## Data Availability

Accession numbers of the raw paired-end reads of all Mtb isolates used in this study are provided in Supplementary Table S2.
