# Supplementary material for: Whole-genome single nucleotide variant phylogenetic analysis of Mycobacterium tuberculosis Lineage 1 in endemic regions of Asia and Africa
Source: Sci Rep. 2022 Jan 28;12:1565. doi: 10.1038/s41598-022-05524-0 (PMC8799649; doi:10.1038/s41598-022-05524-0)
Supplement: Supplementary file 1 — Supplementary Information 1. [file 41598_2022_5524_MOESM1_ESM.pdf]

Supplementary Information for the manuscript

Whole-Genome Single Nucleotide Variant Phylogenetic Analysis of *Mycobacterium tuberculosis* Lineage 1 in endemic regions of Asia and Africa

Thidarat Netikul[a, b], Yuttapong Thawornwattana[b, c], Surakameth Mahasirimongkol[d], Hideki Yanai[e], Htet Myat Win Maung[f, g], Virasakdi Chongsuvivatwong[g], Prasit Palittapongarnpim[b, h, *]

[a)] Faculty of Medicine, Siam University, Phet Kasem Road, Bangkok, Thailand

[b)] Pornchai Matangkasombut Center for Microbial Genomics, Department of Microbiology, Faculty of Science, Mahidol University, Rama 6 road, Bangkok, Thailand

[c)] Department of Organismic and Evolutionary Biology, Harvard University, Cambridge, MA, USA

[d)] Department of Medical Sciences, Ministry of Public Health, Nonthaburi, 11000, Thailand

[e)] Fukujuji Hospital and Research Institute of Tuberculosis, Japan Anti-Tuberculosis Association, Kiyose, 204-8533, Japan

[f)] National TB Control Programme, Department of Public Health, Ministry of Health and Sports, Naypyitaw, 15011, Myanmar

[g)] Epidemiology Unit, Faculty of Medicine, Prince of Songkla University, Had Yai, 90110, Thailand

[h)] National Science and Technology Development Agency, Pathumthani, Thailand

This supplementary PDF file includes the following information:

Supplementary Figures S1-S8

Supplementary Table S1-S8

References to supplementary information

Note: Supplementary Figures S1, S3, and S8 and Supplementary Tables S2-S8 are provided as individual files.

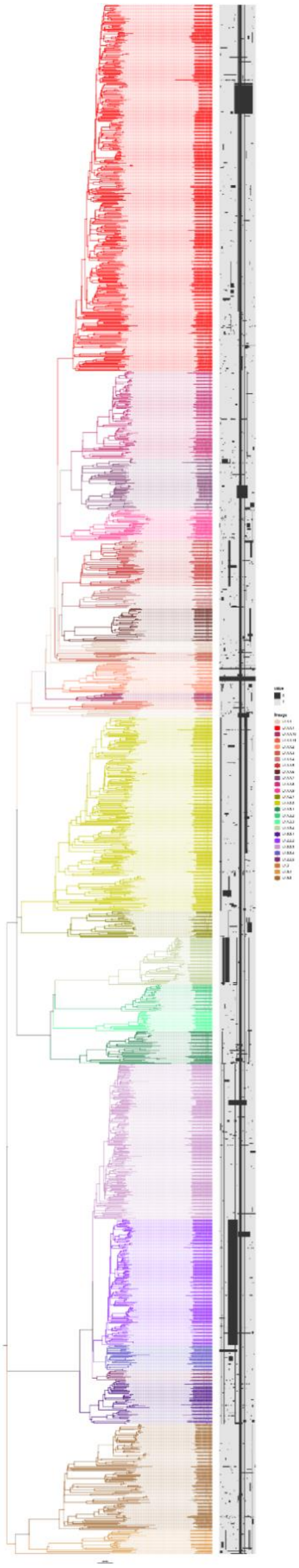

Supplementary Figure S1. A maximum likelihood phylogenetic tree of 1,764 isolates of *M. tuberculosis* lineage 1 colored by sublineages. The diagram on the right shows the deleted DVRs of the CRISPR (Direct Repeat) region of each isolate. The label on the right side of the tree indicates the SRR number, sublineage, country of origin, deleted DVR position and the number of deleted DVRs of each isolate in parenthesis. Every isolate belonging to lineage 1 has deleted DVR39-42, 44 and 48.

Supplementary Figure S1: Netikul Supplementary Figure S1.pdf

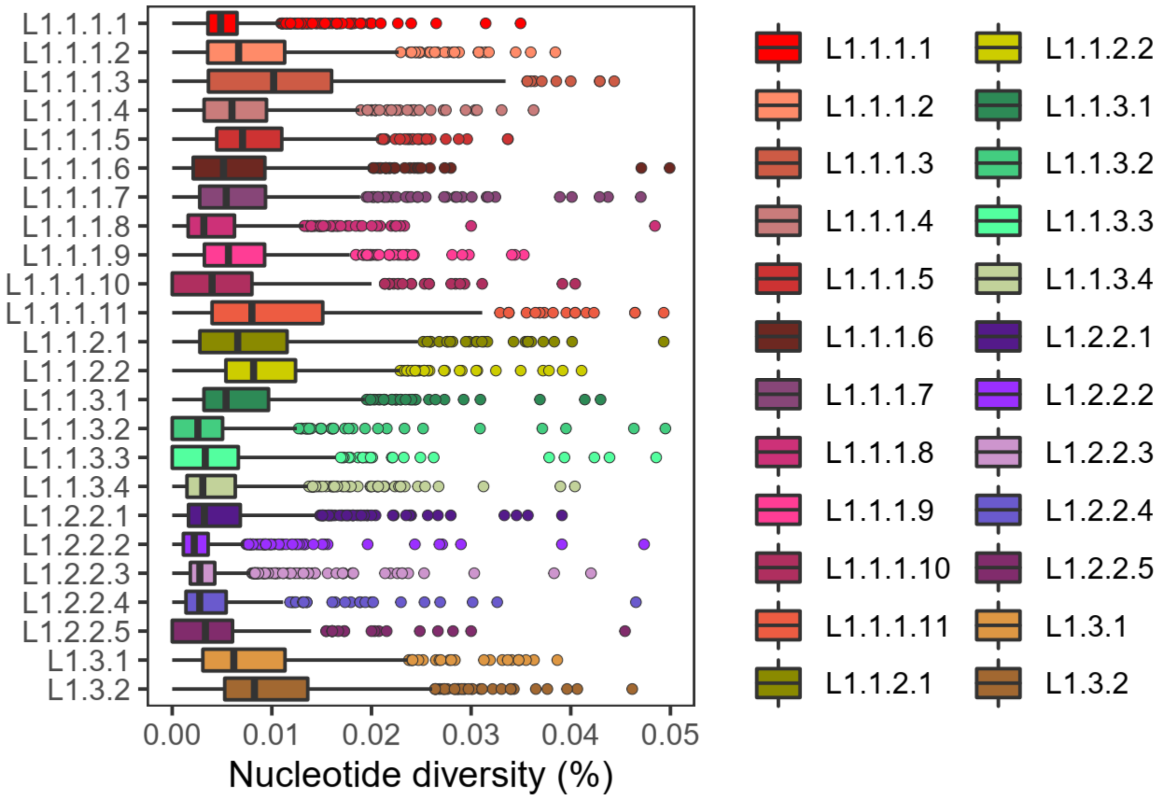

Supplementary Figure S2A

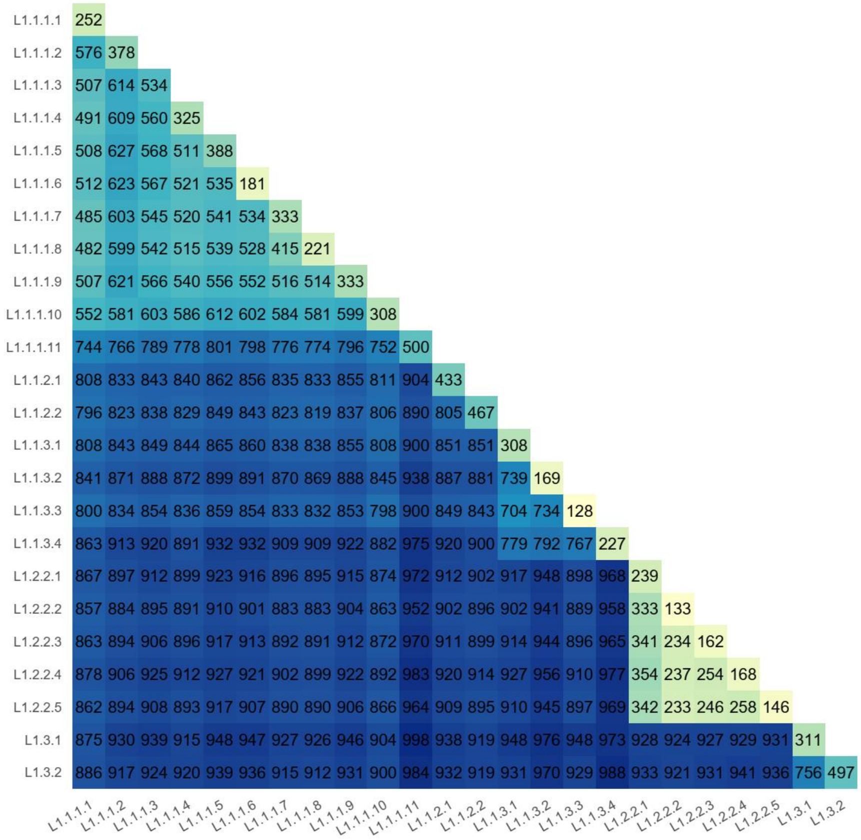

Supplementary Figure S2B

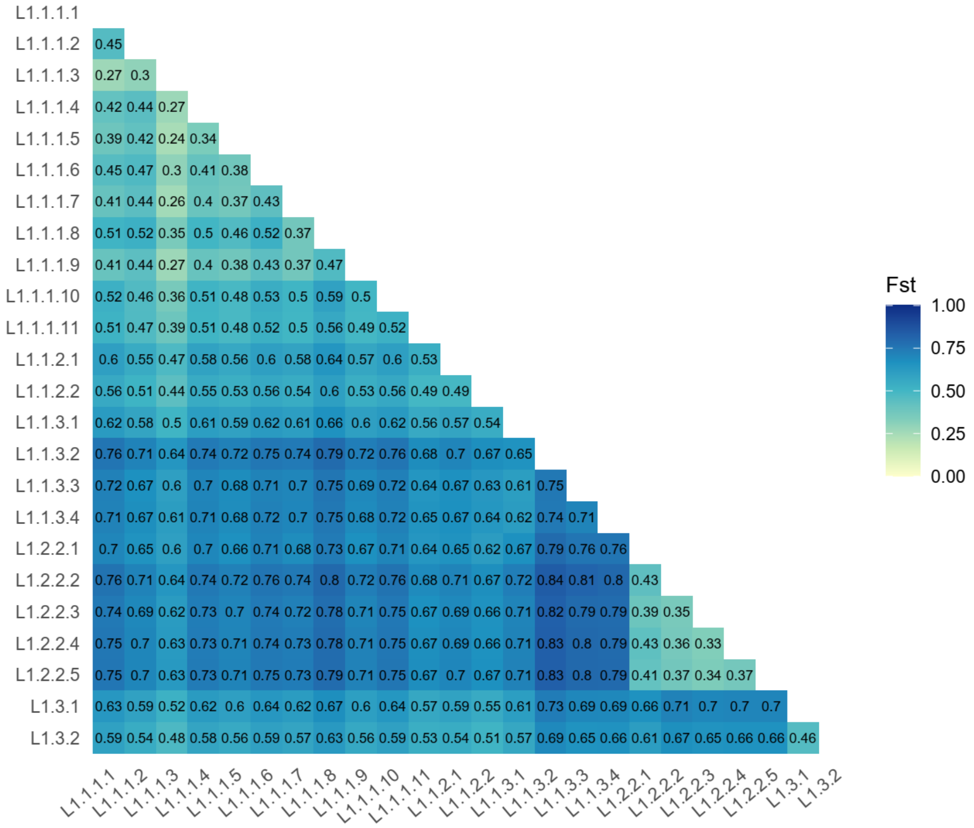

Supplementary Figure S2C

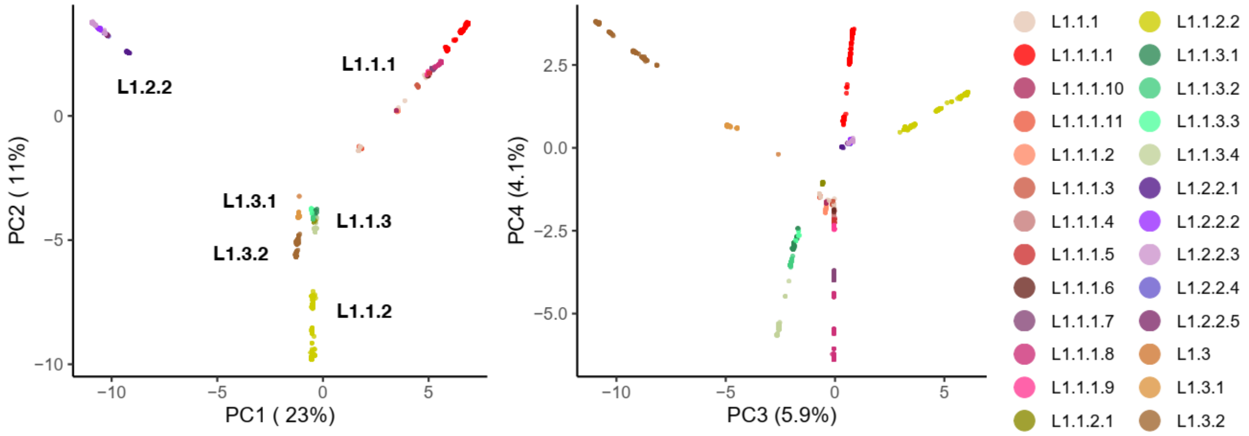

Supplementary Figure S2D

Supplementary Figure S2. Analysis of SNV diversity of 1,764 isolates of *M. tuberculosis* lineage 1. A) The genome-wide averaged nucleotide diversity (π) for each sublineage. B) A table showing average pairwise SNV distances between each pair of sublineages. The numbers in the diagonal indicate the average intra-sublineage SNV distances. C) A table showing fixation index of each pair of sublineages. D) Graphical representations of the results of principal component analysis of the whole genome SNV diversity.

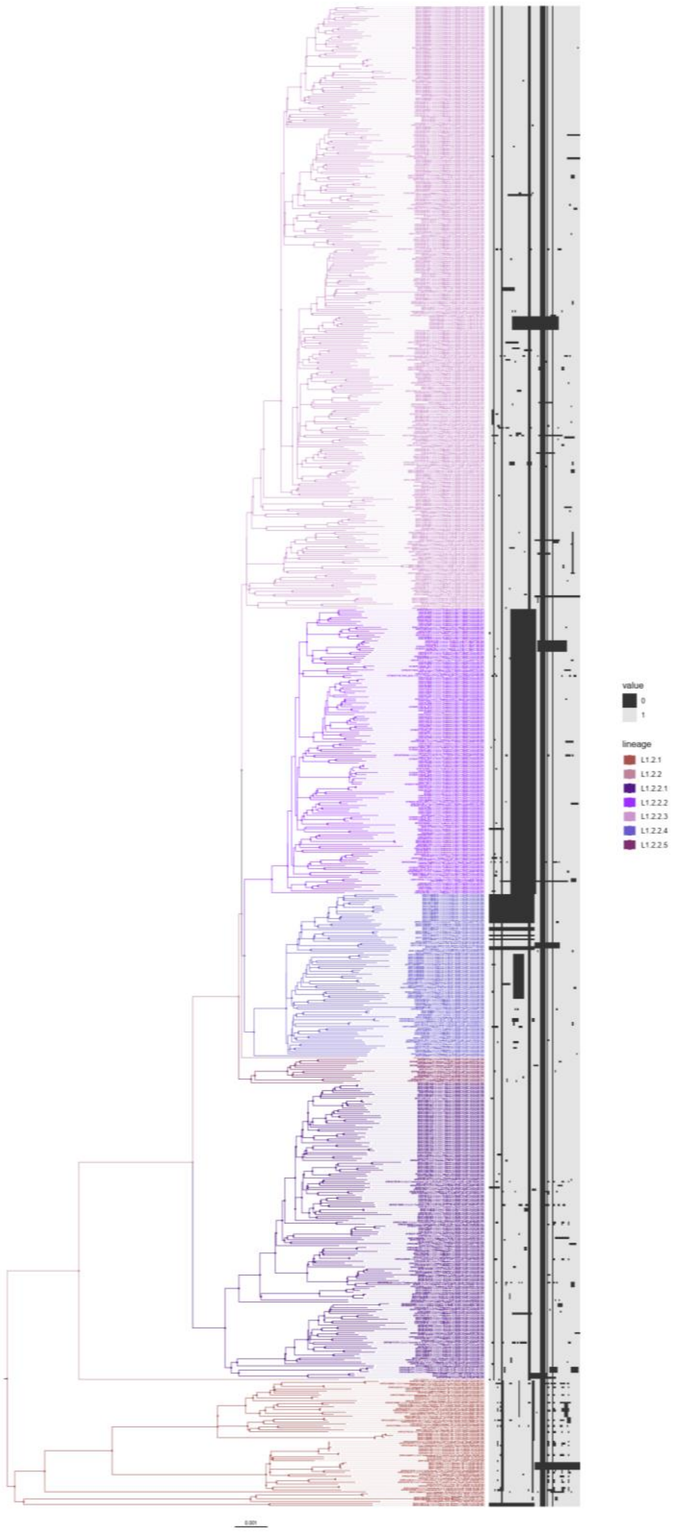

Supplementary Figure S3. A maximum likelihood phylogenetic tree of 774 isolates of *M. tuberculosis* sublineage 1.2 colored by the third- and fourth-leveled sublineages. The diagram on the right shows the deleted DVRs of the CRISPR (Direct Repeat) region of each isolate. The label on the right side of the tree indicates the SRR number, sublineage, country of origin, deleted DVR position and the number of deleted DVRs of each isolate in parenthesis. Every isolate belonging to lineage 1.2 has an additional DVR10 deletion.

Supplementary Figure S3: Netikul Supplementary Figure S3.pdf

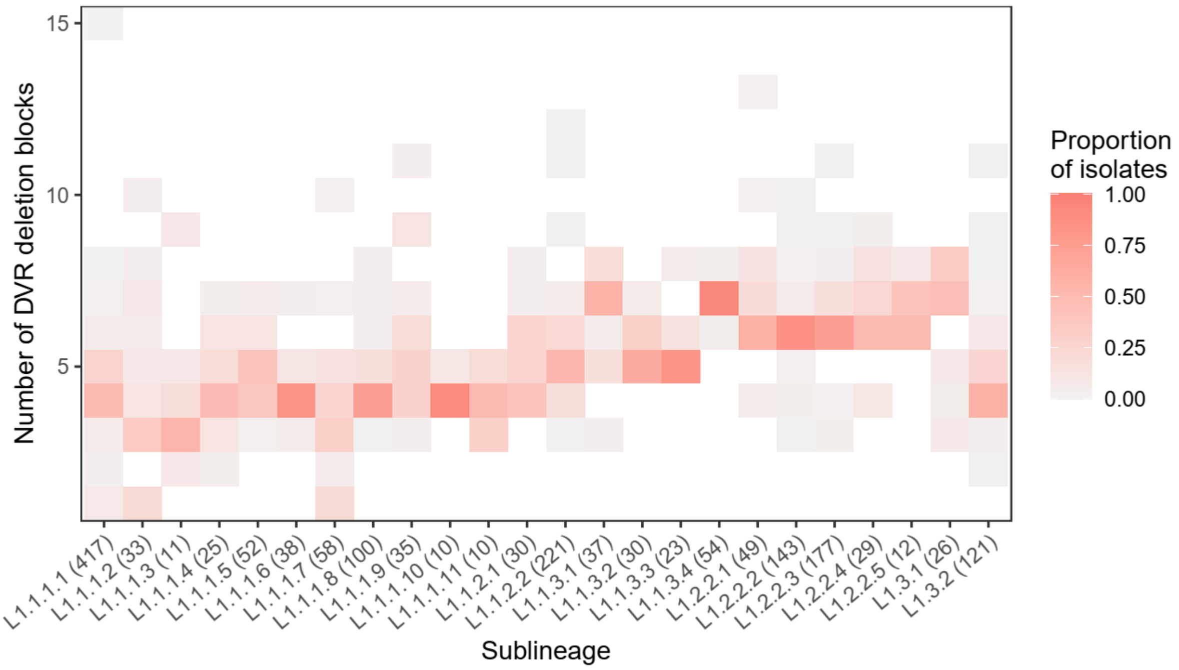

Supplementary Figure S4. A diagram indicating frequencies of minimal numbers of deleted DVR blocks of each sublineage. The number in each parenthesis in the X-axis indicates the number of studied isolates in each sublineage.

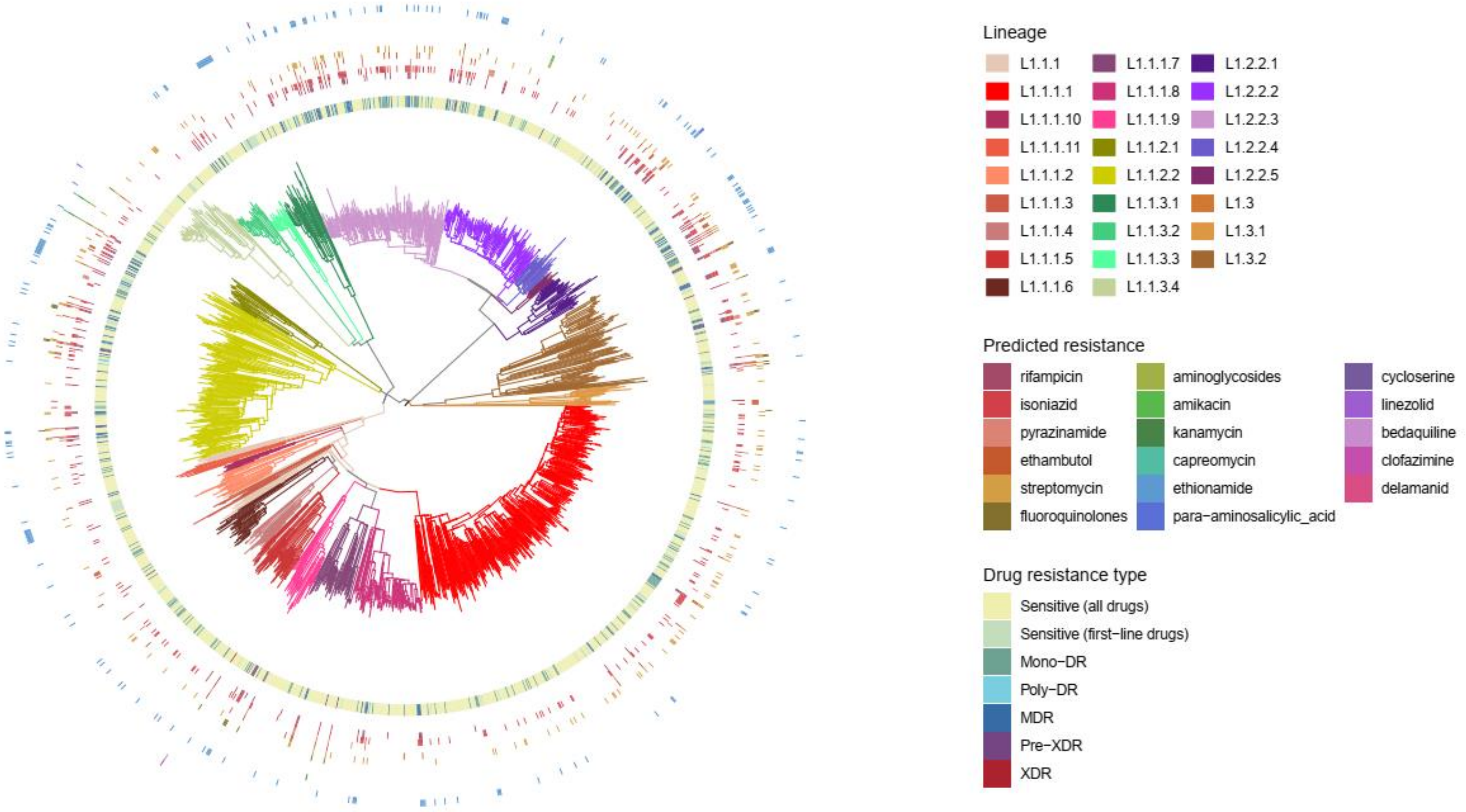

Supplementary Figure S5. A phylogenetic tree of 1,764 isolates of *M. tuberculosis* lineage 1 and predicted drug resistance. The innermost ring indicates the types of drug resistance. The next rings indicate predicted resistance to rifampicin, isoniazid, pyrazinamide, ethambutol, streptomycin, fluroquinolones, and so on, as listed in the legend in the right.

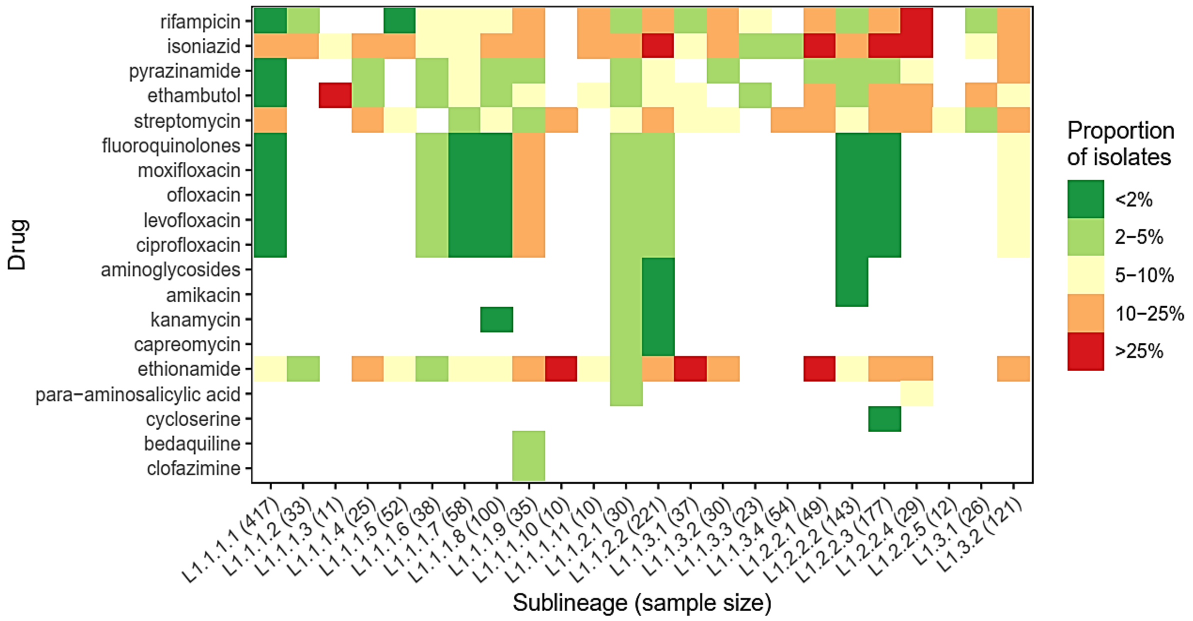

Supplementary Figure S6. A diagram indicating percentages of drug resistance of each sublineage. Uncolored blocks indicate the absence of any drug resistance. The number in the parenthesis in the X-axis indicates the number of studied isolates in each sublineage.

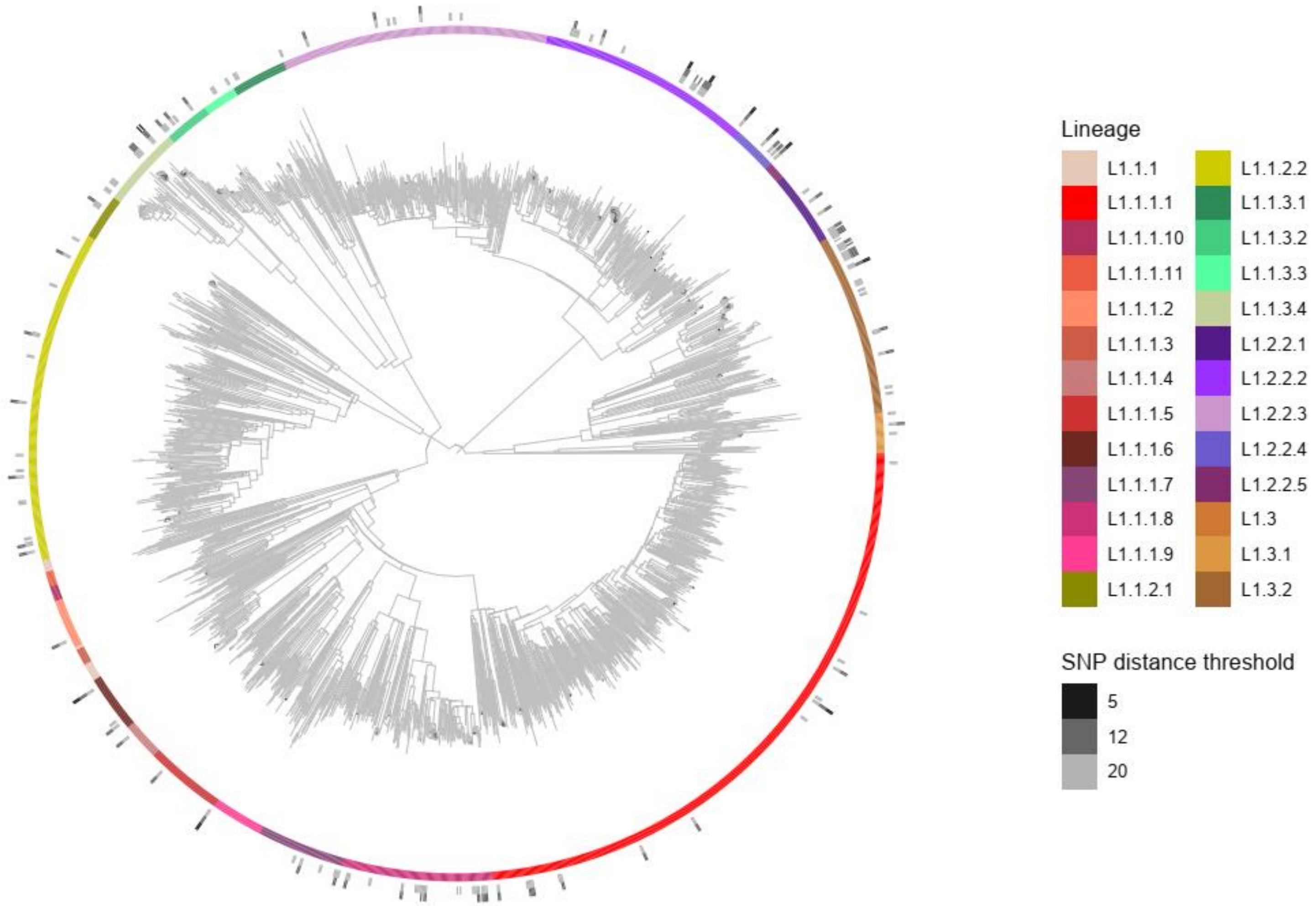

Supplementary Figure S7. A phylogenetic tree of 1,764 isolates of *M. tuberculosis* lineage 1 and genetic clusters based on various SNV difference cut-points. The colored ring indicates the sublineages of the isolates. The genetic clusters are indicated as three rings of bars for each cut-point criterion and also by lines connecting the tips of the tree.

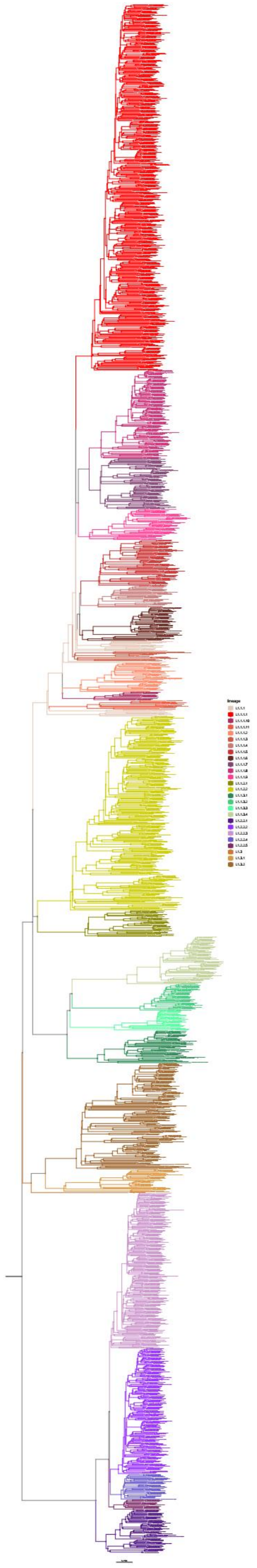

Supplementary Figure S8. Maximum-likelihood phylogeny of 1,764 L1 isolates, correcting for ascertainment bias. The bootstrap supports are consistent with the tree in **Fig. 1** for all sublineages.

Supplementary Figure S8: Netikul Supplementary Figure S8.pdf

Supplementary Table S1. WGS data sources. List of sources of Mtb L1 genome sequence data of 1,764 isolates in the main dataset. Additional 364 L1.2 isolates used in the L1.2 dataset were obtained from three studies: Napier et al. (2020)[1], Menardo et al. (2021)[2] and Bainomugisa et al. (2021)[3].

Region	Country	#isolates*	#L1**	Study accession	Reference
**East Asia**	Japan	191	9	PRJDB7006	Iwamoto et al. (2019)[4]
	Japan	197	13	PRJDB3875	Mizukoshi et al. (2017)[5]
	Taiwan (TB-ARC)	41	4	PRJNA191021	-
	China	306	3	PRJEB23157	Liu et al. (2018)[6]
	China (Guangxi)	358	1	PRJCA002021	Lin et al. (2020)[7]
**Southeast Asia**	Thailand	300	58	PRJNA390471	Faksri et al. (2018)[8]
	Thailand (Chiang Rai 1)	1,188	475	PRJEB7056	Ajawatanawong et al. (2019)[9]
	Thailand (Chiang Rai 2)	658	96	-	-
	Thailand	33	4	PRJNA598981	Nonghanphithak et al. (2020; 2021)[10,11]
	Thailand	29	1	PRJNA598949	Nonghanphithak et al. (2020)[10]
	Malaysia	15	3	PRJNA542351	Tan et al. (2020)[12]
	Malaysia	13	11	PRJNA575111	Jani et al. (2020)[13]
	Myanmar	14	2	PRJEB10037	Aung et al. (2016)[14]
	Myanmar	168	22	-	-
	Vietnam	1,635	381	PRJNA355614	Holt et al. (2018)[15]
	Vietnam	4	1	PRJDB8543	Hang et al. (2019)[16], Maeda et al. (2020)[17]
	Vietnam	80	21	PRJDB8544	Hang et al. (2019)[16]
	Vietnam	248	57	PRJDB8553	Hang et al. (2019)[16], Maeda et al. (2020)[17]
	Philippines	180	143	PRJEB31905	Phelan et al. (2019)[18]
	Philippines	10	6	PRJNA376623	Roa et al. (2018)[19]
	Indonesia	322	8	PRJNA430531	Ruesen et al. (2018)[20]
**South Asia**	India	200	15	PRJNA379070	Advani et al. (2019)[21]
	India	74	4	PRJNA376471	doi:10.1101/847715
	India (TB-ARC)	229	149	PRJNA235852	Manson et al. (2017)[22]
	India	98	65	PRJNA512266	Munir et al. (2019)[23]
	Pakistan	81	3	PRJEB32684	Jabbar et al. (2019)[24]
**Central Asia**	Iran	37	1	PRJNA566379	Kardan-Yamchi et al. (2020)[25]
**Oceania**	Australia	65	8	PRJNA393378	Martinez et al. (2018)[26]
	USA (Hawaii)	85	36	PRJNA480117	Koster et al. (2018)[27]
**East Africa**	Mozambique	138	39	PRJEB27421	Goig et al. (2020)[28]
	Uganda (TB-ARC CDRC)	78	1	PRJNA219826	Manson et al. (2017)[22]
	Malawi	354	58	PRJEB2358	Glynn et al. (2015)[29]
	Uganda	51	1	PRJEB2424	Clark et al. (2013)[30]

Region	Country	#isolates*	#L1**	Study accession	Reference
	Tanzania	88	7	PRJEB29435	Katale et al. (2020)[31]
**Southern Africa**	South Africa (TB-ARC KRITH)	223	6	PRJNA183624	Cohen et al. (2015)[32]
	South Africa (TB-ARC KRITH 2)	217	4	PRJNA235618	Cohen et al. (2015)[32]
	South Africa	43	5	PRJEB9976	Black et al. (2015)[33]
	South Africa	190	2	PRJEB15382	Koch et al. (2017)[34]
**Central Africa**	Republic of the Congo	130	3	PRJEB9545	Malm et al. (2017)[35]
**West Africa**	Liberia	51	10	PRJEB32589	López et al. (2020)[36]
	Sierra Leone	92	4	PRJEB7727	Feuerriegel et al. (2015)[37]
	Mali	74	4	PRJEB27446	Senghore et al. (2020)[38]
**North Africa**	Sudan	166	4	PRJEB33896	Shuaib et al. (2020)[39]
**Multiple**	Total	225	7	PRJEB3223	Comas et al. (2013)[40]
	Asia L1 subset:				
	Vietnam		*4*		
	Africa L1 subset:				
	Uganda		*1*		
	Tanzania		*1*		
	Ghana		*1*		
**Multiple**	Total	127	6	PRJEB11653	Phelan et al. (2016)[41]
	Asia L1 subset:				
	Bangladesh		*3*		
	Philippines		*3*		
**Multiple**	Global reference	21	3	PRJEB27802	Borrell et al. (2019)[42]
	Asia L1 subset:				
	China		*1*		
	India		*1*		
	Philippines		*1*		

NOTES:

* Number of isolates based on the number of BioSample records.

** Number of L1 isolates after excluding samples that failed quality control (see Methods).

Supplementary Table S2. Sample information. Accession numbers for each isolate include study accession ('study'), run accession ('id') and sample accession ('biosample'). 'depth' and 'breadth_d20' show read-mapping statistics which reflect data quality. 'main dataset' and 'L1.2 dataset' indicate isolates included in each of the dataset. Three columns 'spoligotype_dvr', 'spoligotype_spotyping' and 'spoligotype_galru' are spoligotypes predicted by using predicted direct variable repeat (DVR) pattern ('dvr' column), spotyping and galru software, respectively. SIT and spoligotype group ('spo_group') are based on the SITVIT2 database. 'dvr' shows the predicted DVR pattern (see Methods), 'dvr_n_del' is the number of deleted DVR, and 'dvr_del_range' summarizes 'dvr' as blocks of deletions.

Supplementary Table S2: Netikul Supplementary Table S2-S7.xlsx/sheet S2-sample-info

Supplementary Table S3. Distribution of 1,764 isolates in the main dataset by country and sublineage.

Supplementary Table S3: Netikul Supplementary Table S2-S7.xlsx/sheet S3-country-lineage

Supplementary Table S4. Proportions of deleted DVR patterns in each sublineage for all 2,128 isolates in the main and L1.2 datasets. Column descriptions 'n' refers to numbers of samples with each dvr pattern. 'prop' is the proportion of isolates with the dvr pattern in the sublineage. 'dvr_n_del' is the number of deleted DVRs and 'dvr_del' summarizes 'dvr' as blocks of deletions.

Supplementary Table S4: Netikul Supplementary Table S2-S7.xlsx/sheet S4-lineage-dvr-spo

Supplementary Table S5. Numbers of sublineage-specific SNPs derived from joint genotyping of all 2,128 isolates in the main and L1.2 datasets.

Supplementary Table S5: Netikul Supplementary Table S2-S7.xlsx/sheet S5-snp-summary

Supplementary Table S6. Sublineage-specific SNPs identified in this study and proposed barcoding SNPs, also compared with Napier's scheme.

Supplementary Table S6: Netikul Supplementary Table S2-S7.xlsx/sheet S6-snp

Supplementary Table S7. Numbers of isolates with mutations conferring drug resistance

Supplementary Table S7: Netikul Supplementary Table S2-S7.xlsx.sheet S7-drug

Supplementary Table S8. Numbers and percentages of isolates belonging to a genetic cluster in each sublineage. No clusters were identified among unclassified L1.1.1 and L1.3 isolates.

			**cutpoint at 5**		**cutpoint at 12**		**cutpoint at 20**			
**Major Sublineage**	**% clustered**	**Sublineage**	**Number of clusters**	**Number of isolates**	**Number of clusters**	**Number of isolates**	**Number of clusters**	**Number of isolates**	**% clustered at cutpoint 20**	**Numbers of isolates**
L1.1.1	9.6%	L1.1.1.1	1	2	7	16	12	28	6.7%	417
		L1.1.1.2			1	2	1	2	6.1%	33
		L1.1.1.3							0.0%	11
		L1.1.1.4			1	2	1	2	8.0%	25
		L1.1.1.5	1	2	2	4	2	4	7.7%	52
		L1.1.1.6	1	2	2	4	3	6	15.8%	38
		L1.1.1.7			2	4	4	10	17.2%	58
		L1.1.1.8			5	13	6	24	24.0%	100
		L1.1.1.9							0.0%	35
		L1.1.1.10							0.0%	10
		L1.1.1.11							0.0%	10
L1.1.2	13%	L1.1.2.1			1	2	1	4	13.3%	30
		L1.1.2.2			6	12	13	29	13.1%	221
L1.1.3	23%	L1.1.3.1					1	3	8.1%	37
		L1.1.3.2			3	6	4	10	33.3%	30
		L1.1.3.3					2	5	21.7%	23
		L1.1.3.4	1	2	2	7	3	15	27.8%	54
L1.2	15%	L1.2.2.1			4	8	6	13	26.5%	49
		L1.2.2.2	3	6	5	16	8	26	18.2%	143
		L1.2.2.3			3	6	8	16	9.0%	177
		L1.2.2.4	2	4	4	8	4	8	27.6%	29
		L1.2.2.5							0.0%	12
L1.3	31%	L1.3.1			1	2	3	6	23.1%	26
		L1.3.2	1	2	9	20	13	40	32.8%	121
**Total**			**10**	**20**	**58**	**132**	**95**	**251**	**14.4%**	**1,741**

References

1 Napier, G. *et al.* Robust barcoding and identification of Mycobacterium tuberculosis lineages for epidemiological and clinical studies. *Genome Medicine* **12**, 114, doi:10.1186/s13073-020-00817-3 (2020).

2 Menardo, F. *et al.* Local adaptation in populations of Mycobacterium tuberculosis endemic to the Indian Ocean Rim. *F1000Res* **10**, 60-60, doi:10.12688/f1000research.28318.1 (2021).

3 Bainomugisa, A. *et al.* Genomic epidemiology of tuberculosis in eastern Malaysia: insights for strengthening public health responses. *Microb Genom* **7**, doi:10.1099/mgen.0.000573 (2021).

4 Iwamoto, T. *et al.* Overcoming the pitfalls of automatic interpretation of whole genome sequencing data by online tools for the prediction of pyrazinamide resistance in Mycobacterium tuberculosis. *PLoS One* **14**, e0212798, doi:10.1371/journal.pone.0212798 (2019).

5 Mizukoshi, F. *et al.* Genetic diversity of Mycobacterium tuberculosis isolates from Tochigi prefecture, a local region of Japan. *BMC Infectious Diseases* **17**, 365, doi:10.1186/s12879-017-2457-y (2017).

6 Liu, Q. *et al.* China's tuberculosis epidemic stems from historical expansion of four strains of Mycobacterium tuberculosis. *Nature Ecology & Evolution* **2**, 1982-1992, doi:10.1038/s41559-018-0680-6 (2018).

7 Lin, D. *et al.* The geno-spatio analysis of Mycobacterium tuberculosis complex in hot and cold spots of Guangxi, China. *BMC Infectious Diseases* **20**, 462, doi:10.1186/s12879-020-05189-y (2020).

8 Faksri, K. *et al.* Comparative whole-genome sequence analysis of Mycobacterium tuberculosis isolated from tuberculous meningitis and pulmonary tuberculosis patients. *Scientific Reports* **8**, 4910, doi:10.1038/s41598-018-23337-y (2018).

9 Ajawatanawong, P. *et al.* A novel Ancestral Beijing sublineage of Mycobacterium tuberculosis suggests the transition site to Modern Beijing sublineages. *Sci Rep* **9**, 13718, doi:10.1038/s41598-019-50078-3 (2019).

10 Nonghanphithak, D. *et al.* Whole-genome sequence analysis and comparisons between drug-resistance mutations and minimum inhibitory concentrations of Mycobacterium tuberculosis isolates causing M/XDR-TB. *PLoS One* **15**, e0244829, doi:10.1371/journal.pone.0244829 (2020).

11 Nonghanphithak, D. *et al.* Clusters of Drug-Resistant Mycobacterium tuberculosis Detected by Whole-Genome Sequence Analysis of Nationwide Sample, Thailand, 2014-2017. *Emerg Infect Dis* **27**, 813-822, doi:10.3201/eid2703.204364 (2021).

12 Tan, J. L., Simbun, A., Chan, K.-G. & Ngeow, Y. F. Genome sequence analysis of multidrug-resistant Mycobacterium tuberculosis from Malaysia. *Scientific Data* **7**, 135, doi:10.1038/s41597-020-0475-x (2020).

13 Jani, J. *et al.* The whole genome sequence data analyses of a Mycobacterium tuberculosis strain SBH321 isolated in Sabah, Malaysia, belongs to Ural family of Lineage 4. *Data Brief* **33**, 106388, doi:10.1016/j.dib.2020.106388 (2020).

14 Aung, H. L. *et al.* Whole-genome sequencing of multidrug-resistant Mycobacterium tuberculosis isolates from Myanmar. *J Glob Antimicrob Resist* **6**, 113-117, doi:10.1016/j.jgar.2016.04.008 (2016).

15 Holt, K. E. *et al.* Frequent transmission of the Mycobacterium tuberculosis Beijing lineage and positive selection for the EsxW Beijing variant in Vietnam. *Nat Genet* **50**, 849-856, doi:10.1038/s41588-018-0117-9 (2018).

16 Hang, N. T. L. *et al.* Whole genome sequencing, analyses of drug resistance-conferring mutations, and correlation with transmission of Mycobacterium tuberculosis carrying katG-S315T in Hanoi, Vietnam. *Scientific reports* **9**, 15354-15354, doi:10.1038/s41598-019-51812-7 (2019).

17 Maeda, S. *et al.* Genotyping of Mycobacterium tuberculosis spreading in Hanoi, Vietnam using conventional and whole genome sequencing methods. *Infection, Genetics and Evolution* **78**, 104107, doi:https://doi.org/10.1016/j.meegid.2019.104107 (2020).
18 Phelan, J. E. *et al.* Mycobacterium tuberculosis whole genome sequencing provides insights into the Manila strain and drug-resistance mutations in the Philippines. *Sci Rep* **9**, 9305, doi:10.1038/s41598-019-45566-5 (2019).
19 Roa, M. B. *et al.* Whole-genome sequencing and single nucleotide polymorphisms in multidrug-resistant clinical isolates of Mycobacterium tuberculosis from the Philippines. *Journal of Global Antimicrobial Resistance* **15**, 239-245, doi:https://doi.org/10.1016/j.jgar.2018.08.009 (2018).
20 Ruesen, C. *et al.* Large-scale genomic analysis shows association between homoplastic genetic variation in Mycobacterium tuberculosis genes and meningeal or pulmonary tuberculosis. *BMC Genomics* **19**, 122, doi:10.1186/s12864-018-4498-z (2018).
21 Advani, J. *et al.* Whole Genome Sequencing of Mycobacterium tuberculosis Clinical Isolates From India Reveals Genetic Heterogeneity and Region-Specific Variations That Might Affect Drug Susceptibility. *Front Microbiol* **10**, 309, doi:10.3389/fmicb.2019.00309 (2019).
22 Manson, A. L. *et al.* Genomic analysis of globally diverse Mycobacterium tuberculosis strains provides insights into the emergence and spread of multidrug resistance. *Nature Genetics* **49**, 395-402, doi:10.1038/ng.3767 (2017).
23 Munir, A. *et al.* Identification and Characterization of Genetic Determinants of Isoniazid and Rifampicin Resistance in Mycobacterium tuberculosis in Southern India. *Scientific reports* **9**, 10283-10283, doi:10.1038/s41598-019-46756-x (2019).
24 Jabbar, A. *et al.* Whole genome sequencing of drug resistant Mycobacterium tuberculosis isolates from a high burden tuberculosis region of North West Pakistan. *Sci Rep* **9**, 14996, doi:10.1038/s41598-019-51562-6 (2019).
25 Kardan-Yamchi, J. *et al.* Whole Genome Sequencing Results Associated with Minimum Inhibitory Concentrations of 14 Anti-Tuberculosis Drugs among Rifampicin-Resistant Isolates of Mycobacterium Tuberculosis from Iran. *J Clin Med* **9**, doi:10.3390/jcm9020465 (2020).
26 Martinez, E. *et al.* Mutations associated with in vitro resistance to bedaquiline in Mycobacterium tuberculosis isolates in Australia. *Tuberculosis (Edinb)* **111**, 31-34, doi:10.1016/j.tube.2018.04.007 (2018).
27 Koster, K. *et al.* Whole genome SNP analysis suggests unique virulence factor differences of the Beijing and Manila families of Mycobacterium tuberculosis found in Hawaii. *PLoS One* **13**, e0201146, doi:10.1371/journal.pone.0201146 (2018).
28 Goig, G. A., Blanco, S., Garcia-Basteiro, A. L. & Comas, I. Contaminant DNA in bacterial sequencing experiments is a major source of false genetic variability. *BMC Biol* **18**, 24, doi:10.1186/s12915-020-0748-z (2020).
29 Glynn, J. R. *et al.* Whole Genome Sequencing Shows a Low Proportion of Tuberculosis Disease Is Attributable to Known Close Contacts in Rural Malawi. *PLoS One* **10**, e0132840, doi:10.1371/journal.pone.0132840 (2015).
30 Clark, T. G. *et al.* Elucidating emergence and transmission of multidrug-resistant tuberculosis in treatment experienced patients by whole genome sequencing. *PLoS One* **8**, e83012, doi:10.1371/journal.pone.0083012 (2013).
31 Katale, B. Z. *et al.* Whole genome sequencing of Mycobacterium tuberculosis isolates and clinical outcomes of patients treated for multidrug-resistant tuberculosis in Tanzania. *BMC Genomics* **21**, 174, doi:10.1186/s12864-020-6577-1 (2020).
32 Cohen, K. A. *et al.* Evolution of Extensively Drug-Resistant Tuberculosis over Four Decades: Whole Genome Sequencing and Dating Analysis of Mycobacterium tuberculosis Isolates from KwaZulu-Natal. *PLoS Med* **12**, e1001880, doi:10.1371/journal.pmed.1001880 (2015).

33 Black, P. A. *et al.* Whole genome sequencing reveals genomic heterogeneity and antibiotic purification in Mycobacterium tuberculosis isolates. *BMC Genomics* **16**, 857, doi:10.1186/s12864-015-2067-2 (2015).
34 Koch, A. S. *et al.* The Influence of HIV on the Evolution of Mycobacterium tuberculosis. *Mol Biol Evol* **34**, 1654-1668, doi:10.1093/molbev/msx107 (2017).
35 Malm, S. *et al.* New Mycobacterium tuberculosis Complex Sublineage, Brazzaville, Congo. *Emerg Infect Dis* **23**, 423-429, doi:10.3201/eid2303.160679 (2017).
36 López, M. G. *et al.* Tuberculosis in Liberia: high multidrug-resistance burden, transmission and diversity modelled by multiple importation events. *Microb Genom* **6**, doi:10.1099/mgen.0.000325 (2020).
37 Feuerriegel, S. *et al.* PhyResSE: a Web Tool Delineating Mycobacterium tuberculosis Antibiotic Resistance and Lineage from Whole-Genome Sequencing Data. *J Clin Microbiol* **53**, 1908-1914, doi:10.1128/jcm.00025-15 (2015).
38 Senghore, M. *et al.* Evolution of Mycobacterium tuberculosis complex lineages and their role in an emerging threat of multidrug resistant tuberculosis in Bamako, Mali. *Sci Rep* **10**, 327, doi:10.1038/s41598-019-56001-0 (2020).
39 Shuaib, Y. A. *et al.* Mycobacterium tuberculosis Complex Lineage 3 as Causative Agent of Pulmonary Tuberculosis, Eastern Sudan(1). *Emerg Infect Dis* **26**, 427-436, doi:10.3201/eid2603.191145 (2020).
40 Comas, I. *et al.* Out-of-Africa migration and Neolithic coexpansion of Mycobacterium tuberculosis with modern humans. *Nat Genet* **45**, 1176-1182, doi:10.1038/ng.2744 (2013).
41 Phelan, J. *et al.* Mycobacterium tuberculosis whole genome sequencing and protein structure modelling provides insights into anti-tuberculosis drug resistance. *BMC Med* **14**, 31, doi:10.1186/s12916-016-0575-9 (2016).
42 Borrell, S. *et al.* Reference set of Mycobacterium tuberculosis clinical strains: A tool for research and product development. *PLoS One* **14**, e0214088, doi:10.1371/journal.pone.0214088 (2019).
