# Supplementary figures and images for: Whole-genome single nucleotide variant phylogenetic analysis of Mycobacterium tuberculosis Lineage 1 in endemic regions of Asia and Africa
Source: Sci Rep. 2022 Jan 28;12:1565. doi: 10.1038/s41598-022-05524-0 (PMC8799649; doi:10.1038/s41598-022-05524-0)

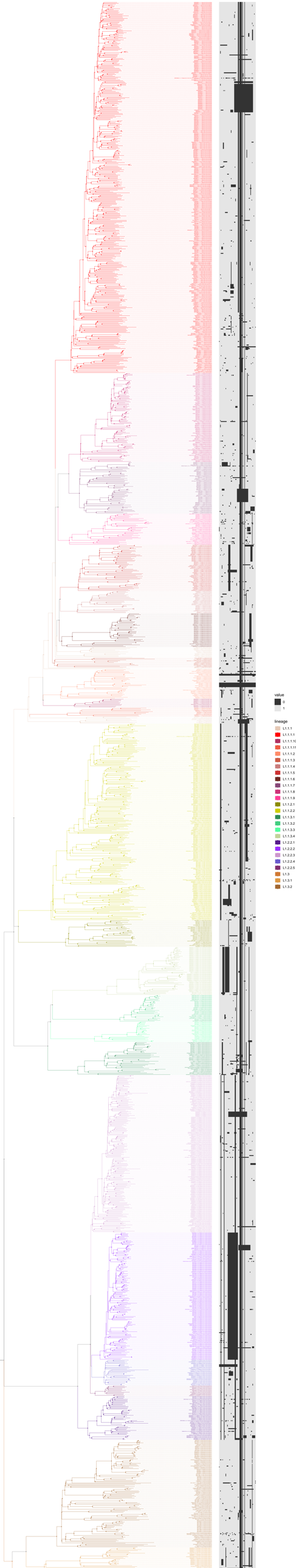

Supplement: Supplementary file 2 — Supplementary Figure 1. [file 41598_2022_5524_MOESM2_ESM.pdf]

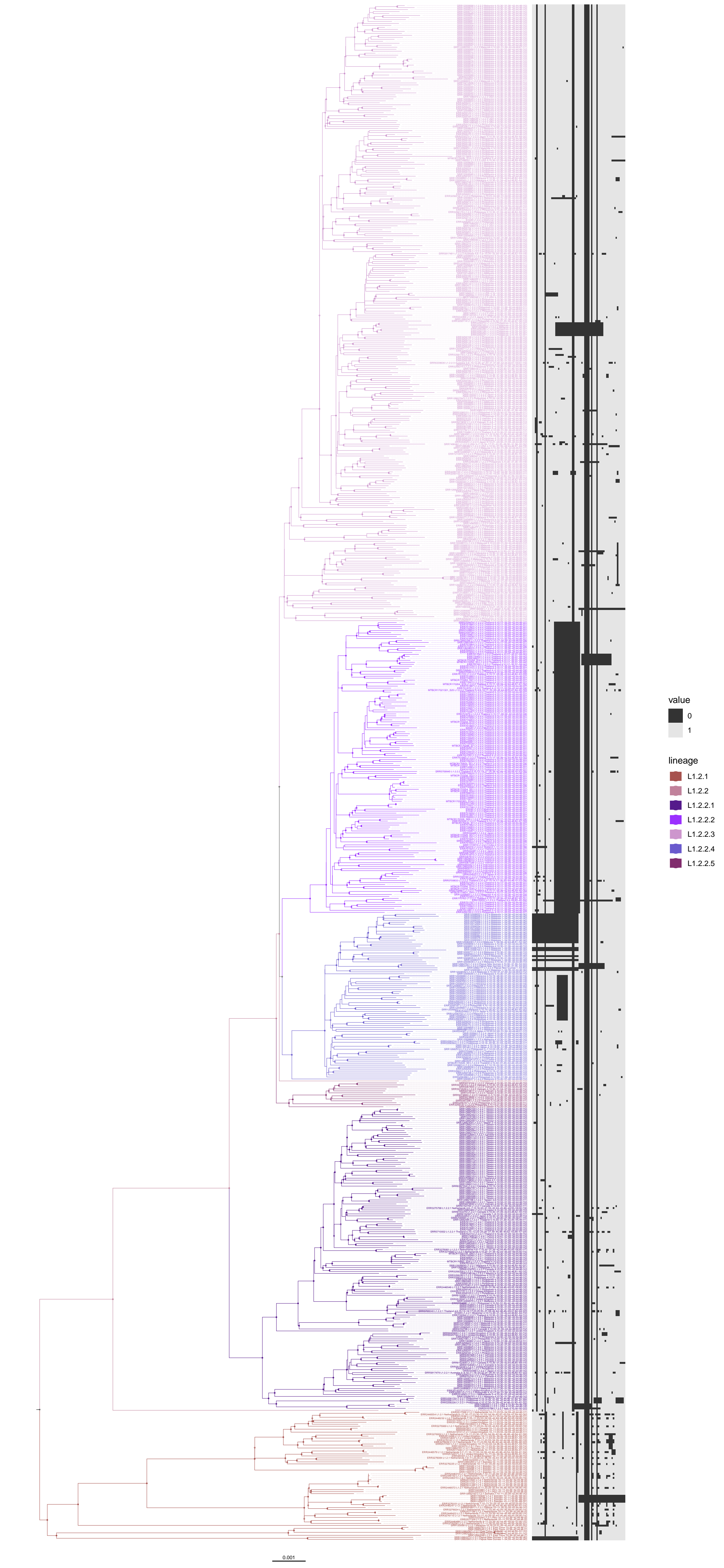

Supplement: Supplementary file 3 — Supplementary Figure 3. [file 41598_2022_5524_MOESM3_ESM.pdf]

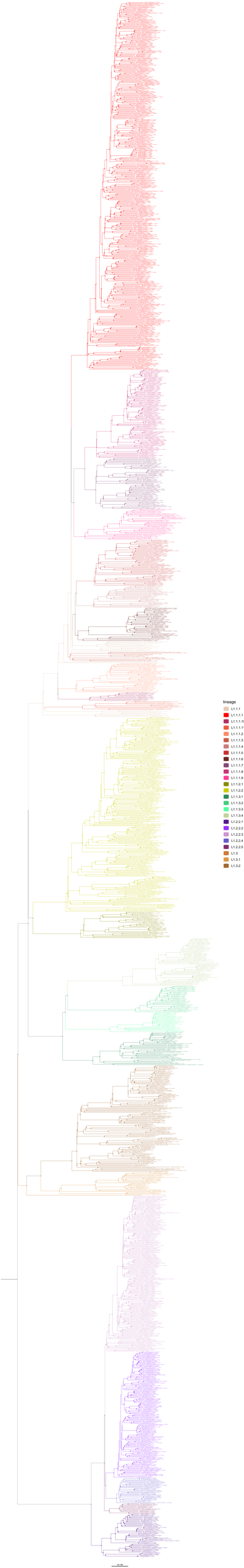

Supplement: Supplementary file 4 — Supplementary Figure 8. [file 41598_2022_5524_MOESM4_ESM.pdf]
